# Quantifying the ambient population using hourly population footfall data and an agent-based model of daily mobility

**DOI:** 10.1007/s10707-019-00346-1

**Published:** 2019-04-27

**Authors:** Tomas Crols, Nick Malleson

**Affiliations:** 1 Environmental Modelling Unit, Flemish Institute for Technological Research – VITO, Boeretang 200, 2400 Mol, Belgium; 2 School of Geography, University of Leeds, Leeds, LS2 9JT UK; 3 Leeds Institute for Data Analytics, University of Leeds, Leeds, LS2 9JT UK

**Keywords:** Agent-based modelling, Ambient population, Big data, Urban analytics, Urban simulation

## Abstract

The ambient population, i.e. the demographics and volume of people in a particular location throughout the day, has been studied less than the night-time residential population. Although the spatio-temporal behaviour of some groups, such as *commuters*, are captured in sources such as population censuses, much less is known about groups such as retired people who have less documented behaviour patterns. This paper uses agent-based modelling to disaggregate some ambient population data to estimate the size and demographics of the constituent populations during the day. This is accomplished by first building a model of *commuters* to model typical *9–5* workday patterns. The differences between the model outputs and real footfall data (the *error*) can be an indication of the contributions that other groups make to the overall footfall. The research then iteratively simulates a wider range of demographic groups, maximising the correspondence between the model and data at each stage. An application of this methodology to the town centre of Otley, West Yorkshire, UK, is presented. Ultimately this approach could lead to a better understanding about how town- and city-centres are used by residents and visitors, contributing useful information in a situation where raw data on the populations do not exist.

**Electronic supplementary material:**

The online version of this article (10.1007/s10707-019-00346-1) contains supplementary material, which is available to authorized users.

## Introduction and background

The ambient (*day-time*) population is of considerable interest. Knowing the locations of people throughout the day is essential for fields such as crime science [1–3], health (i.e. exposure to air pollution) [4–7], events management [8], and many others. The ambient population is also being modelled to get a better understanding of the mobility of urban individuals [9]. A range of modelling approaches, including agent-based models (ABMs), already provide insight into the residential (*night-time*) population, but estimates of the ambient population are much more scarce. However, the emergence of *smart cities* and the associated *data deluge* has led to a diverse range of new data sources that could potentially be used to better understand ambient populations. The rise of these *big* data streams therefore presents an opportunity to improve the calibration and validation of geographical simulation models in urban areas [10, 11]. Possible datasets include, for example, footfall data (i.e. through CCTV cameras or Wi-Fi sensors), transport smart card data, mobile phone usage and social media location data. We argue here that ABMs of daily mobility can be used to identify the spatio-temporal locations of high concentrations of people and the nature of their activities [12] and, ultimately, improve estimates of the ambient population and our understanding about which demographic groups of people use urban spaces at different times.

In recent years a few individual-based models have been developed to track the ambient population [e.g. 4, 13, 14], but these have not yet used all of the benefits offered by agent-based modelling and have not focused on any particular groups of people. Some groups, such as the young or elderly, might be more vulnerable to phenomena such as crime [15] and environmental pollution [16, 17], or in greater need of good public transport connections [18]. These vulnerabilities are coupled with a lack of data on the typical routines of such groups, especially in comparison to those employed with typical *9–5* jobs who have reasonably regular commuting times and destinations, the details of which are available from sources such as population censuses. For example, current research on elderly people in a smart cities context is typically about tracking individual people who wear sensors – often with the aim of being able to locate them in case of an emergency [19, 20] – but not about the location of the group as a whole throughout the day.

**Fig. 1:**
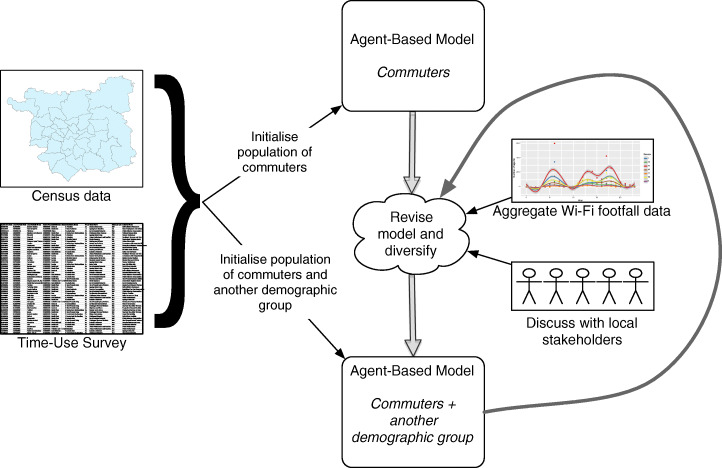
The iterative model development process

The aim of this work is to explore the efficacy of agent-based modelling as a means of simulating the daily spatio-temporal behaviour of different population groups to better understand how town- and city-centres are used during the daytime. An iterative approach, as illustrated by Fig. 1, is adopted. This process will be explained briefly here and elaborated on in detail later. The model begins by simulating the behaviour of groups of people who are the most extensively documented in the available data. In this case, that group are *commuters*, for whom both the residential and workplace locations are known from the national population census. The census data are combined with a time use survey and an agent-based model is created to represent the average daily behaviour of people within this group. The accuracy of the model is then assessed against an aggregate spatio-temporal measure of footfall. In this case, sensors that detect smart-phone Wi-Fi connections are used to estimate the number of people in certain locations at certain times. Note that these data do not distinguish individuals; only an aggregate count per unit time is available. Initially there is an inevitable large difference between the real footfall data and those being simulated because the model only represents one broad group of people, whereas the sensor data include everyone (or at least those with a mobile phone; a point that will be revisited later). The modelling process then proceeds to iteratively increases the number of demographic groups who are being modelled in order to gradually improve the correspondence between the model and the available data.

Agent-based models of daily routines are typically based on the daily schedules of agents within households. The MATSIM model [21] has an excellent transport component in which travel times can be optimised, but has also been criticised because travel times are not optimal in reality, and the survey-based agent schedules in the model are rather static [13]. TRANSIMS is another well-known model of the mobility of agents and their daily activity plans [22, 23] but suffers similar criticisms. Several other travel demand models of scheduled daily commuter behaviour have been developed [e.g. 13, 14, 24, 25], including dynamic approaches with evolving households [e.g. 13, 14]. The model presented in this article (hitherto entitled the *Simulating Urban Flows (surf)* model) differs from the approaches mentioned above in that it does not use daily schedules to determine agent behaviour: agents change from one activity to another based on the intensity of their motivation to do a specific activity. Intensities reflect the goals that the agents want to achieve first [26] (i.e. the activity with the highest motivation intensity is the one that is conducted). This approach was chosen in order to allow for greater flexibility in the schedules of the agents – the research outlined here is part of a broader programme of work that ultimately aims to calibrate models dynamically based on streams of current ambient data, so a more dynamic approach to scheduling and behaviour was judged as more appropriate than static schedules.

The study area is Otley, a town in West Yorkshire, UK, and its suburban surroundings, and the chosen demographic groups are commuters and retired people. The model calibration is based on census data of commuting flows and home locations of retired people, and on the results of the UK Time Use Survey 2014–2015 [27]. The following sections of the paper discuss the study area, the census and footfall data, the time use survey, and the development of the *surf* model. After that, the results section presents the observed differences between the total ambient population (as measured using observed data) and the modelled demographic groups. Finally, the current methodology is discussed and new ideas to improve the model and its data, along with methods for calibration and validation, are proposed.

The paper is structured as follows. Section 2 outlines the study area and the available data. Section 3 then discusses the model itself, with reference to the ODD+D description that is available in the appendix. Section 4 outlines the process of iteratively improving the model by including a greater diversity of agent types. Section 5 then presents the results and the paper concludes with a discussion and conclusion in Sections 6 and 7.

## Study area and data

### Otley and the census data

**Fig. 2:**
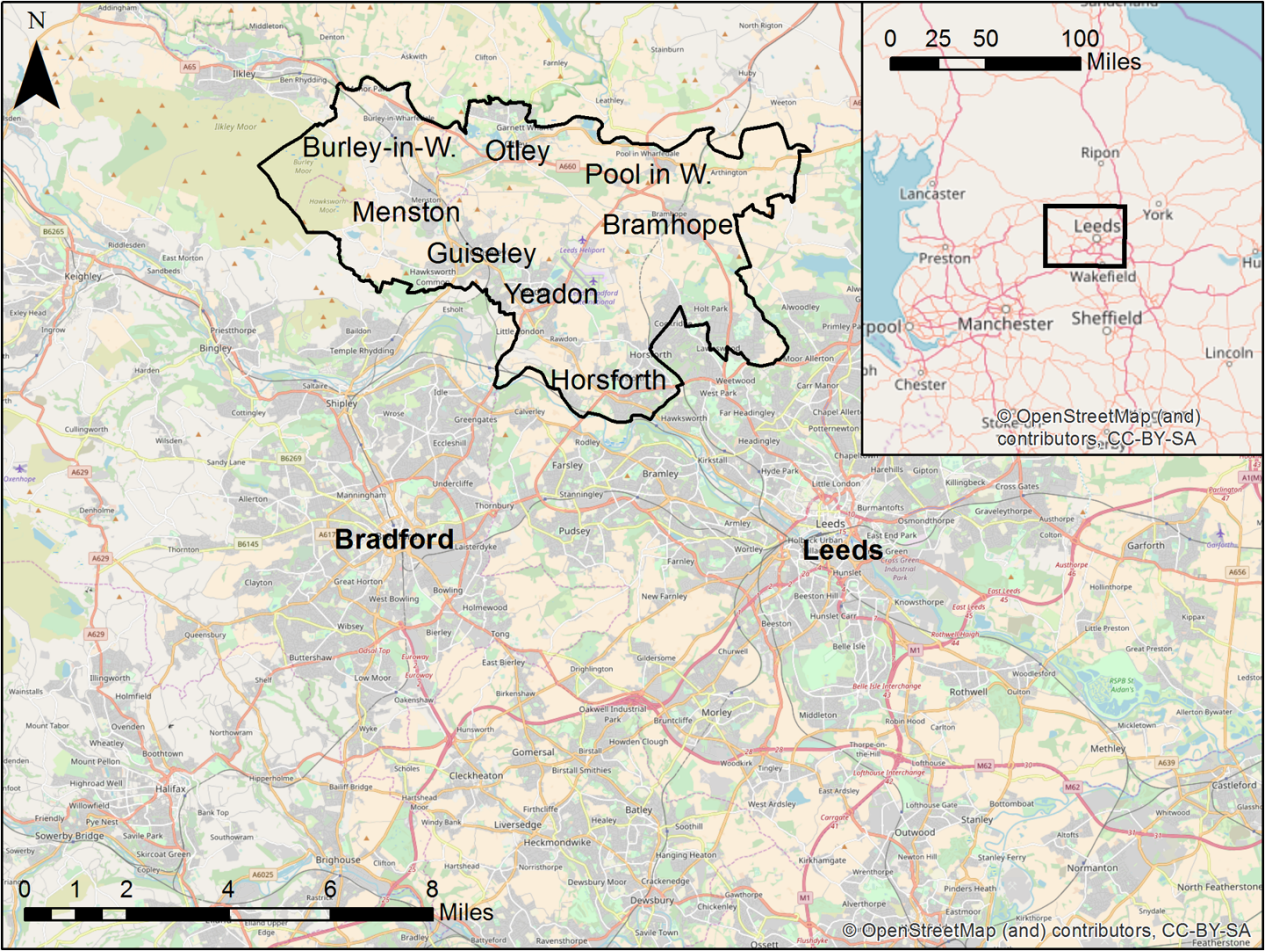
Map of the study area and the cities of Leeds and Bradford, and position of this area in northern England

The study area is centred on the market town Otley in the Leeds Metropolitan District, West Yorkshire, UK (Fig. 2). Otley was chosen for two reasons: firstly it has a relatively self-contained commuting pattern (the majority of the people who work in Otley do not travel far); and secondly because there is an excellent source of high-resolution spatio-temporal data from a number of Wi-Fi sensors that can be used to quantify the real footfall patterns in the town. The aim of the model is to simulate the behaviours of commuters and retired people in Otley. Therefore a study area must be drawn that includes people who live and work (if they work) either in Otley or in the surrounding area. Commuting data were obtained from the 2011 UK Census [28] and contain flows of people from residential to employment locations at the level of output areas (OAs), the smallest statistical unit in the UK. The data were used to identify all of the people who commuted into or within Otley, and the study area was then constructed to include the residential locations of the majority of this group. A very small number of people commuted from outside of this area (some from hundreds of miles away) but these were not included. The study area includes Otley and a few surrounding towns (Horsforth, Yeadon, Guiseley, Menston, Burley-in-Wharfedale, Pool in Wharfedale and Bramhope). The census data contains the number of people who commute between all combinations of OAs in the study area. As such, there are 11,807 commuting agents in 773 OAs. The number of retired people per OA was also extracted from the 2011 census, leading to 32,535 retired agents.

To accompany the census data, footfall data from eight Wi-Fi sensors in the centre of Otley were provided for by the commercial organisation ‘Noggin’ and the Otley Business Improvement District (a public organisation whose role is to further the economic potential of the town). The sensors capture the signals of Wi-Fi enabled mobile devices of pedestrians. Sensors next to main roads and especially crossroads can also detect car passengers during periods of congestion, when their mobile devices stay near the sensor for a sufficient amount of time. The dataset contains hourly totals for each location which were collected between August 2015 and July 2017.

### The UK time use survey

In order to model the population to the greatest degree of accuracy, the most recent UK Time Use Survey (UKTUS) was used to better understand the activities of commuters and retired people. The UKTUS 2014–2015 [27, 29] contains daily activities of 8278 respondents in 4238 households, after eliminating incomplete records. It is a follow-up of an earlier large-scale survey conducted in 2000–2001, and it is the British part of the Multinational Time Use Survey (MTUS) [30]. The respondents fill in diaries per 10 min interval during two days. For every interval, they specify which main activity they were doing at which type of location (at home, at work, in a shop, on the road, etc.). Additionally, they can mention secondary activities, other people who were with them, a level of enjoyment of the activity, and whether they were using a computer, tablet or smartphone. The survey contains the ceremonial county in which the households live. Due to the limited number of respondents in West Yorkshire (only 268), data for the entire UK were used. There appears to be a bias with new activities starting on the (half) hour rather than on other 10 min intervals. This is probably because the survey is retrospective; respondents do not fill in their diaries on the spot. This issue is not problematic here since as such relatively small biases will not influence the overall model results.

**Fig. 3:**
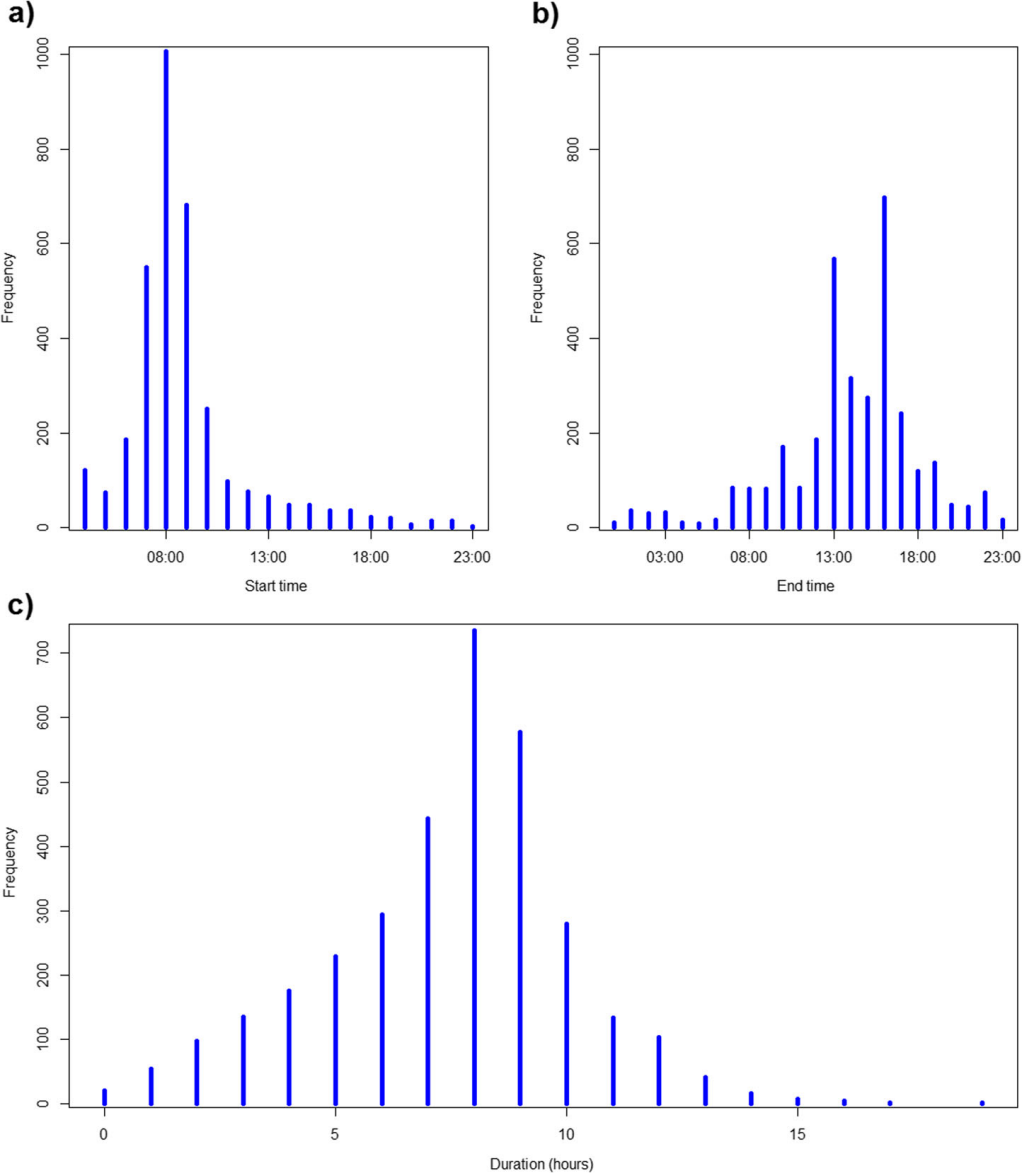
Statistics of working schedules in office buildings in the UK in the UKTUS [27]: the distribution of (**a**) start times and (**b**) end times with hh:00 meaning the time interval [hh:00, hh:59], and (**c**) duration of a workday in the office rounded to the nearest full hour

An understanding of the activities that drive peoples’ daily travel behaviour, as well as the approximate start times and durations, is essential for identifying the most important activities to include in the agent-based model. The analysis focused on finding the proportion of respondents (either commuters or retired people) doing away-from-home activities on weekdays, and specifically workdays for commuters (i.e. excluding weekends and all days on which the commuters do not work). Subsequently, the start and end time frequency and average duration of these away-from-home activities were extracted. Many commuters start their day at the office between 07:00 and 10:00, and remain there on average for 8 h (Fig. 3). Part-time working usually takes place in the morning as only very few people start working after lunch. With regard to all other activities of both groups, shopping, sports and social activities are the most popular. At-home activities (household and family care, mass media, hobbies at home) were excluded since no travelling is involved.

**Fig. 4:**
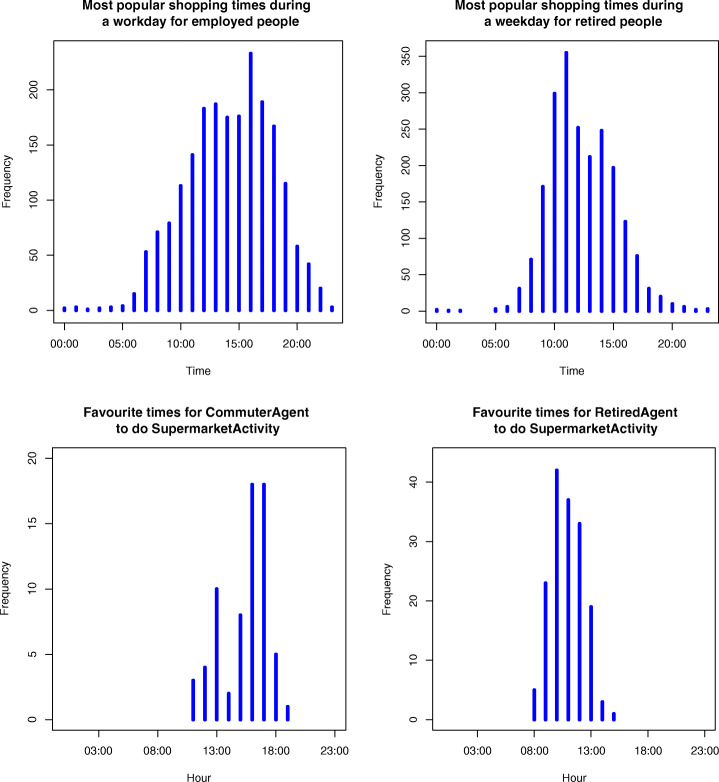
Statistics of shopping for food by commuters and retired people in the UK in the UKTUS [27] (top) versus model results for commuter and retired agents after calibration (bottom)

Around 27% of all commuters go shopping for food on an average workday (43% on Friday). Shopping is more popular during lunchtime and the afternoon rush hour (Fig. 4), and the duration is typically short with a mean time of 41 min but a median of only 30 min. More retired people go shopping for food on weekdays (49%), and spend on average 69 min in the supermarket (with a median of 50 min). Mornings are slightly preferred over afternoons (Fig. 4). Leisure shopping is done by 16.6% of retired people per day, and can best be handled as a separate activity.

The next most popular activity is sports, with 16.2% of commuters and 17.8% of retired people participating. A smaller, but not negligible, proportion of commuters go for lunch (4.5%) or do a social food or drink evening activity (7.9%). Similar percentages can be found for retired people for lunch not at home (7.9%) and social evening activities (7.9% as well). The analysis of this survey indicates that to model both groups, the most important activities, other than *being at home* and *working*, are *shopping*, *sports*, *having lunch*, and *going out for food and drinks*. The following section discusses the model itself and outlines how the results of the time use survey were used to parameterise the agents’ behaviour such that their activities corresponded with the appropriate times as identified in the survey (e.g. as in Fig. 4 for shopping).

## The *surf* model

### Agent behaviour

The *Simulating Urban Flows (surf)* model represents the daily routines and travel patterns of individual *9–5 commuter* agents and *retired* agents on weekdays. Analysis of the time use survey highlighted seven different activities that need to be modelled in the first instance (later iterations of the model can continue to add activities as required): being at home, working in an office (*commuters* only), shopping for food in supermarkets, leisure shopping (*retired* only), having lunch (if not at home or at work), going out in the evening for food and/or drinks, and doing sports. The behaviour of the agents has been structured to respond to these activities.

Each activity has an *intensity* – a motivation to do this activity – and the activity with the highest intensity at any given time is the one that drives an agent’s behaviour (although there is also a *minimal duration* for the present activity that prevents agents rapidly changing activities). The *total intensity* is the sum of a *background intensity* and a *time intensity*, which differ by each activity.

The *background intensity* reflects the recurrence pattern. It gradually increases when the agent is not performing the activity, and decreases during the activity. The rate of increments and decrements are different for each activity and can be configured. After calibration, the rates should be as such that the typical recurrence and duration of activities are correct, as extracted from the UKTUS (i.e. the agents spend an appropriate amount of time, as per the time use survey, on each activity). For example, the survey suggests that, on average, a commuter goes to the supermarket about every 3 days and spends about 30 min shopping. The rate that the background intensity increases can be configured so that the right proportion of agents go shopping on each day, and the rate that it decreases while an agent is shopping can be configured so that agents spend, on average, 30 min on that activity. Of course this will vary on a daily basis as other activities might be more important on a particular day, such that the agent might not shop at all for 3 days, but then spend more time shopping on the next day. The rate of decrease in background intensity is always more rapid than the increases, such that agents can always perform an activity in order to reduce its intensity back to 0. This is especially true for activities that typically have short time periods (e.g. less than an hour of shopping can reduce a shopping intensity that has been building up over several days).

The *time intensity* is also activity-specific, reflecting common times when agents do that activity. For example, the time intensity for *being at home* becomes stronger over night. These times are also based on the UKTUS. Stochasticity is included in both intensity types to create heterogeneous agents. The result of the manual calibration of intensities should be that average activity statistics per hour of the day should be very similar to those observed in the UKTUS. As can be seen in Fig. 4 for shopping for food, the pattern of the model results is similar to reality, though with less variability or outliers. The main reason for the more limited variability is that time intensity was set to zero outside the most important time interval, which vastly improved the pattern of that time interval.

**Fig. 5:**
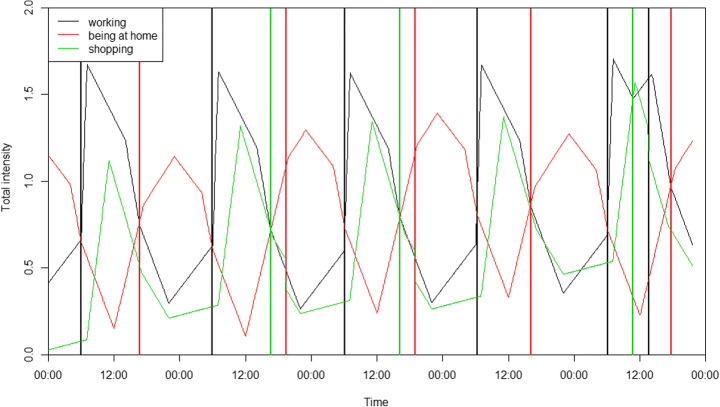
Total intensities of three activities (being at home, working, shopping) of one agent in the model during 5 days. The agent starts at home and vertical lines in different colours indicate when new activities start

A diagram of a simplified example of an agent only being at home, working and shopping with the changing activities and total intensities during 5 days is given in Fig. 5. The agent begins at home and vertical lines indicate when new activities start. The *total intensity* can still increase after the start of an activity when the *time intensity* is going up (e.g. working in the early morning), but the *background intensity* will decrease nevertheless. This agent goes to work in the morning and returns home in the afternoon at varying times. On days 1 and 4, the intensity for shopping is never sufficiently high, meaning that the agent does not do this activity on those days. On days 2 and 3, the agent goes shopping after work, while they go during their lunch break on day 5. This shows that the intensities indeed make it possible to have flexible agent behaviour.

For the spatial allocation of flexible activities, it is assumed that it is more likely that agents will do these nearby the location of their previous activity. Data about the functions of buildings are extracted from OpenStreetMap, and activities are allocated to random buildings with the correct function within a search radius of the previous activity. This search radius is iteratively doubled if necessary.

Details on agent behaviour, scheduling, initialisation, and the model environment can be found in the ODD+D description in the Online appendix.

### Implementation

The model is coded in Scala, but makes use of the Java-based multi-agent simulation library MASON [31] and its geographical toolkit GeoMASON [32]. The agents travel through the study area at a constant speed, approximately equal to walking speed, so the model currently does not account for different road types or congestion. The shortest path between activities is computed with the A* algorithm of GeoMASON. The time step is five minutes and the model is typically run for 14 days. Although the model reaches equilibrium after approximately the first two days, only the results of the final day of simulation are used. The models is also executed 50 times to account for its probabilistic nature and the average results across all model runs are reported – i.e. the graphs that report the number of agents who pass each sensor in a particular hour actually show the *mean* number of agents across all model runs. As the following section will evidence, there is little variability in the separate model runs.

## Iterative model improvements through diversification and validation

The final aim of this work is to better understand how a town centre is used at different times of day. Data on *total* hourly footfall are available for the study area, but these counts are not disaggregated by the demographics of the individual groups who contribute to the total. The spatio-temporal behaviour of some groups, such as *commuters*, can be estimated from available data (such as population censuses that include details about peoples’ living and working locations) but this is not the case for others such as people who are retired and will not regularly travel to a work place. To simulate these groups, for which little or no local empirical data are available, this work iteratively expands and diversifies the range of demographic groups who are simulated, maximising the correspondence between the model and available footfall data at each stage. This allows us to disentangle the contributions that different groups make to the overall footfall at different times of day.

This approach presents a problem regarding validation. Typically, the model would be calibrated using one dataset and validated using another. In this case, however, no validation data are available. Indeed, if such data were available then the agent-based model would be unnecessary as we would already have a reasonable understanding about how the town centre was used. Hence there is a need to question the suitability of empirical validation in this case, and to attempt to develop alternative means. It has been argued, very convincingly in some cases [33], that it is *impossible* to validate numerical models of open systems in the first place. This is because the system will be influenced by external factors (otherwise it would not be an ‘open’ system) and a model would thus require input parameters that were not known. Indeed, if a model were able to reproduce real-world empirical data from an open system exactly, then it would also be able to account for these unknown external parameters; a situation that would be “suspicious” [34]. Furthermore, complex systems can undergo “regime shifts” [35], where the system shifts into a radically different trajectory. The validation data might therefore represent one particular trajectory at the expense of others, leading the process of empirical validation to unintentionally constrain the model to one particular trajectory. Similarly, if the data represent a particularly unlikely trajectory then the more general model might fail to validate.

Ultimately, it can be argued that “validation by fit-to-data is not necessarily (on its own) a helpful measure in complex systems” [34]. This is relevant here because, short of conducting a resource-intensive survey of individuals in the study area (which is beyond the scope of the project), there are no additional data that can be used to validate the model. That is not to say, however, that empirical validation is unnecessary. It can be a useful benchmarking process to estimate the “empirical adequacy” of a model [33]. The question is, instead, *how* the model could be validated in an alternative to “fit-to-data”.

One potential approach is to leverage the *ontological structure* that is inherent in agent-based models. Unlike with most other modelling approaches^1^ (e.g. regression or neural networks) the structure of the agent-based model can reveal something about the underlying system. Therefore, there is value in validating the *ontology* of an agent-based model, as well as validating its outcomes [34]. This approach is like using “expert involvement” to validate the structure of the model – Hassan et al. [36] present detailed recommendations for effectively using expert advice. Here, however, there is limited value in reviewing the behavioural routines of the agents (e.g. the intensity functions) as these are not supposed to reflect *real* human decision making. Instead, the preliminary modelling *results* were presented to stakeholders who were familiar with the case study area. This presented an opportunity to allow face validation from locals who could provide guidance about how well the model was performing, and how it could be improved. It needs to be recognised, of course, that local opinions will be skewed and biased, but in the absence of more rigorous data they can be valuable.

**Fig. 6:**
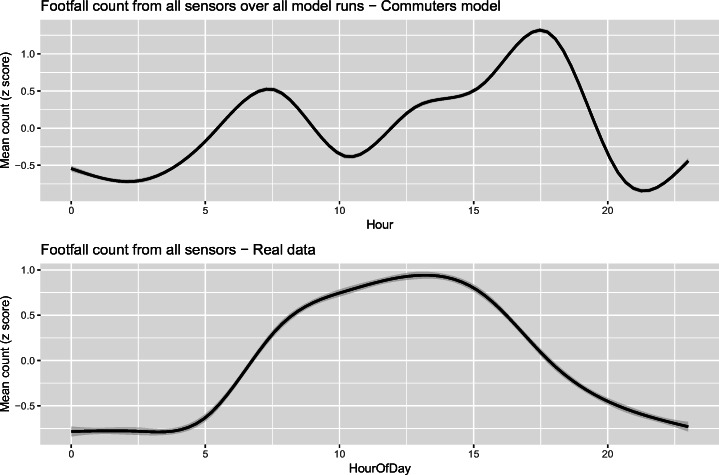
The mean footfall recorded at all sensor locations in the model across 50 independent model runs with 99% bootstrap confidence intervals (these are very narrow and difficult to distinguish). The line of best fit is produced using GAM

To this end, two events were organised: (1) the work was presented to a mixture of local residents and policy makers at an “Otley Science Café”; (2) individual meetings were set up with local experts from the Otley Business Improvement District.^2^ Both groups were presented with the results of an early iteration of the model that only included commuters and were asked to explain the differences between the simulated results and the real data. Fig. 1 broadly outlined this process of iterative improvement and Fig. 6 (in the results section) presents the real data and simulated results that were shown to the stakeholders. The groups agreed that the results did successfully represent the behaviour of commuters and highlighted some reasons for the possible inconsistencies. In particular, four main groups were identified as being absent in the model but occasionally present in large numbers in the town:

1. Retired people who might take grandchildren to school and attend the town after the morning rush hour;
2. Parents who take children to school and then perform other activities (shopping, going to cafés and attending sports classes were mentioned);
3. Tourists who come in from outside of the study area, particularly for the market or during cycle events (although the predominance of these groups on non-market days during the week is questionable);
4. Some children (aged 14–16) from the local school who are allowed to leave the school grounds between 13:10–13:50 to eat lunch (approx. 500 students) and are likely to be registered by at least two of the sensors.

The results of these meetings served two purposes. Firstly, they performed a useful form of validation in that the results of the *commuting* model were largely in agreement with the expectations of the stakeholders. Secondly, the results highlighted the groups that were most obviously absent. Census data also confirmed that retired people are an overrepresented group in the Otley area, in comparison with the rest of Leeds and Bradford, and in some of the OAs surrounding Otley centre in particular. Both the stakeholder conclusions and the census data were part of the motivation for including a new agent type to represent *retired people* in the next iteration of the research. Future iterations can continue this process of inclusion and refinement.

## Preliminary results

The iterative nature of the methodology used here requires the results from each stage to be presented consecutively. The *commuting* model is discussed in Section 5.1 and the clear discrepancies between the model results are real footfall data are highlighted. This version of the model was the one that the stakeholders discussed and commented on. Section 5.2 then presents the results of the model that includes *retired people*. Finally, Section 5.3 presents the footfall by individual sensor. The results are discussed as a whole in Section 6. Recall that the *real* footfall data consist of counts of Wi-Fi enabled smartphones that are sensed as their owners pass by a sensor location. To allow for direct comparisons between the simulated and real-world datasets, similar counts were taken in the model (i.e. every time an agent passed one of the sensor locations in the virtual environment, a counter for that sensor was incremented). The following results will compare the footfall produced by the model and compare it to the *real* data collected from Otley.

### Results of the *commuting* model

Figure 6 illustrates the total footfall in the model and compares it to that from the real sensor data. For the simulated data (top), the chart shows the mean footfall across all sensors at each hour of the simulation in the last 24-h period of the final day (to ensure equilibrium has been reached), averaged across all of the 50 separate model runs. The results are depicted with a smooth line generated using a generalised additive model (GAM) [37] as implemented in the *mgcy* R package. Although 99% boot strap confidence intervals have been included in the figure, these are so narrow as to be nearly impossible to discern, which is encouraging as it demonstrates that each model run produces similar aggregate results. Note that the apparent increase in simulated footfall (top) after 20:00 is an artefact of the GAM line smoothing algorithm. For the real sensor data (bottom), the graph shows the mean footfall across all sensors on Tuesdays, Wednesdays and Thursdays (to capture ‘normal weekday’ behaviour). Note that for both charts Z-scores are used to make the scales visually comparable.

With respect to the simulated activity patterns, there are two activity peaks of particular note. The first occurs in the morning; it is caused by agents travelling from their homes to their workplaces (i.e. the morning rush hour). There follows a reduction in footfall as the agents are at work. Later, there is a gradual increase in activity as some agents go to lunch or shopping, leading to the most substantial peak in the afternoon when they commute home or start other activities after work. These patterns are entirely expected given the way that the model has been parameterised based on the evidence available about daytime activities in the time use survey. In effect, these patterns are a representation of the *expected* behaviour patterns of commuters who travel in to work in Otley. The differences between the model outputs and real footfall data (the *error*) can be partially explained by the contributions that other groups make to the overall footfall. Obviously, there are other possible sources of error, e.g.: (1) the spatial and temporal uncertainty of the data used, (2) changing behaviour of the population between the census (2011) and the validation period (2015–2017), or (3) the spatial allocation and the routing without congestion in the model. After discussing the results with local stakeholders, the model was extended to include a second group of agents, who could be expected to constitute a large part of the error: those representing *retired people*.

### Results of the *commuting & retired* model

**Fig. 7:**
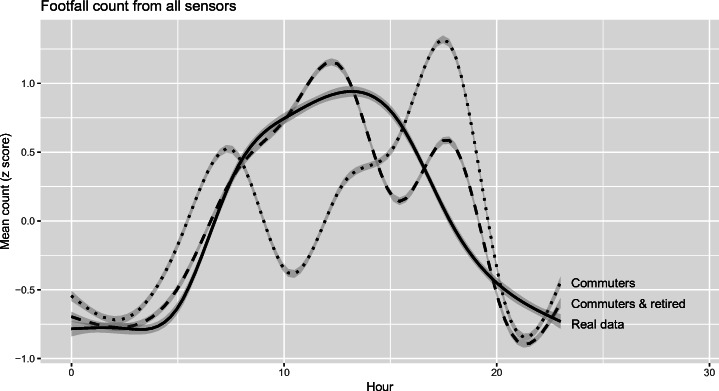
Comparing simulated (dashed and dotted lines) and real-world (solid lines) hourly footfall data. The fit lines were produced using GAM

Figure 7 illustrates the results of the *commuting* model, the *commuting & retired* model, and the real sensor data. Again Z-scores are used to scale the results to comparable ranges. The results of the updated model are much more similar to those of the real footfall data. The substantial drop in footfall that was being simulated at around 10:00 disappears as retired agents begin their activities. Whilst this is not validation, the success of this incremental addition to the model gives some more confidence that the results can reveal some insight into the dynamics of the case study area. This is information that, before now, was not available other than in entirely aggregate form. Interestingly, the most substantial difference between the model and real data (error) now occurs around 15:00. It is very likely that this error is partially caused by the substantial body of children who leave the various schools at 15:00; they (and their carers for younger children) are therefore a prime target for inclusion in further iterations of the work.

### Results by individual sensor

**Fig. 8:**
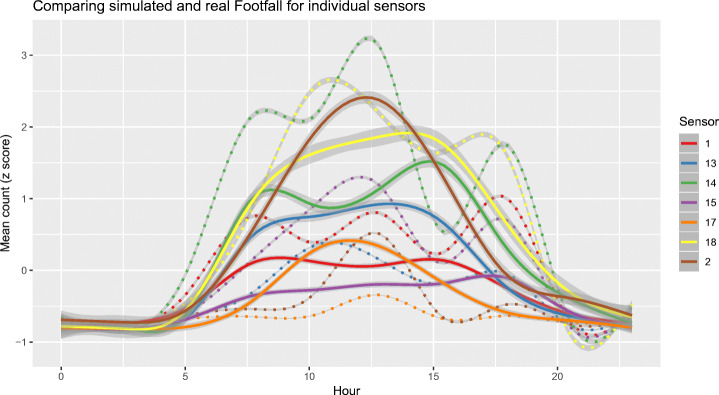
The mean footfall recorded at all sensor locations in the real data (solid lines) and model (dotted lines) across 50 independent model runs with 99% bootstrap confidence intervals (these are very narrow and difficult to distinguish). The line of best fit is produced using GAM

**Fig. 9:**
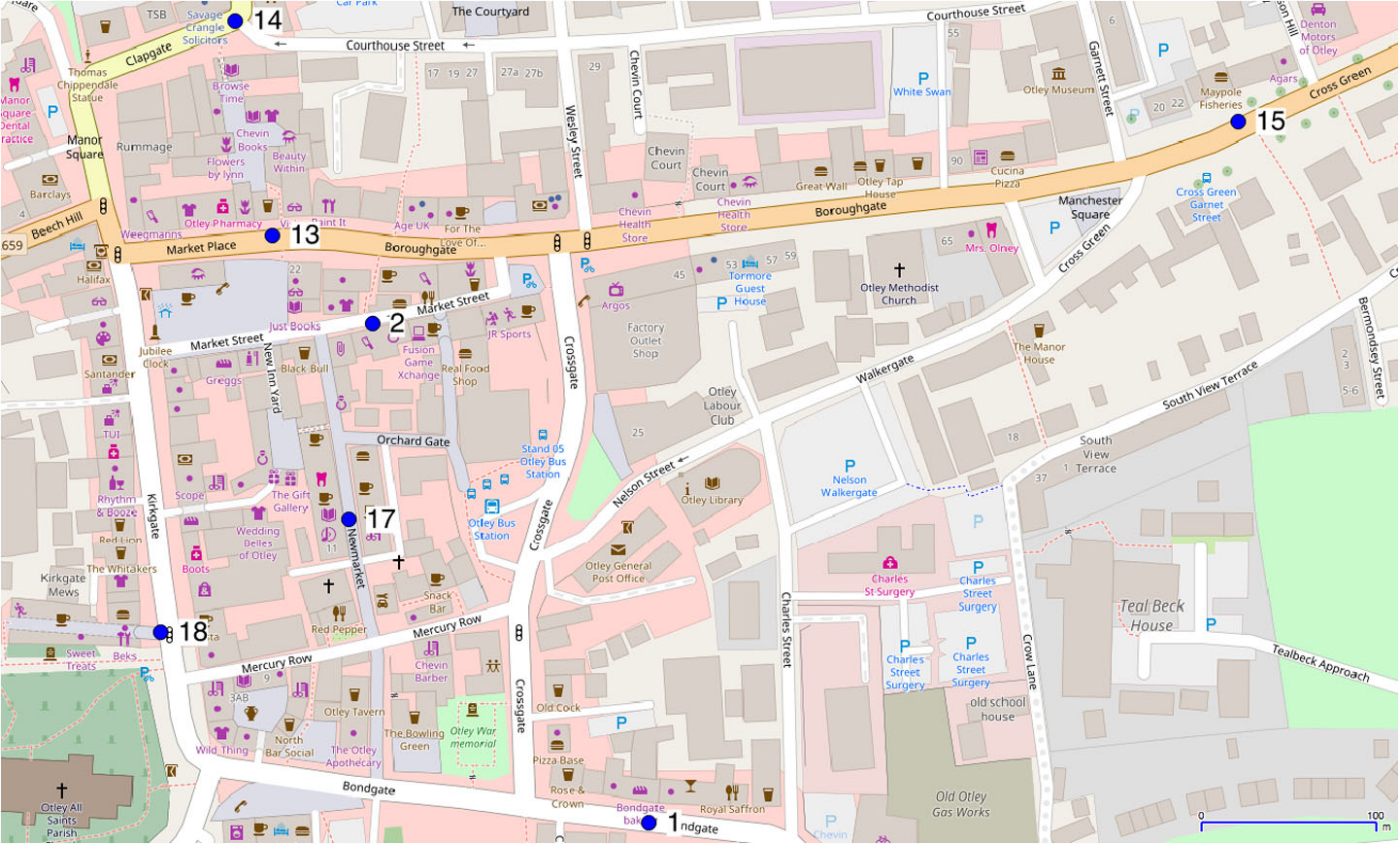
The location of each sensor in the centre of Otley

Finally, Fig. 8 illustrates the footfall broken down by the individual sensor (see Fig. 9 for a map of the locations). The sensors were installed in the first place to give an average picture of footfall across the whole town, as has been done so until this point. But it is nevertheless interesting to review some of the possible spatial differences across sensors. Sensors 1 and 14 are both placed near to the arterial routes into the town, whereas the others are all within the main town centre. The arterial sensors are more likely to be on routes used by commuters, and less likely to catch the leisure activities of people in the town during the day. It is encouraging to find rush hour peaks in both the sensor data and the model results for locations 1 and 14, even though the exact times are slightly different, and the model has lunch-time peaks too.

## Discussion

The aim of this work, as outlined in the introduction, has been to explore the efficacy of agent-based modelling as a means of simulating the daily spatio-temporal behaviour of different population groups to better understand how town- and city-centres are used during the daytime. This section will review the preliminary findings and discuss the extent to which this aim has been met. Later sections then outline the main caveats and present ideas for immediate future work.

### Preliminary findings

The activities of some groups of people are easier to estimate than for others. For example, from the 2011 UK census there is an abundance of data about where people live and, for employed people, where they go to work. By coupling these data with estimates of the timings of their activities – in this case from a large time use survey – it is possible to estimate the spatio-temporal activities of a reasonably large group of people. Here we call them *commuters*. The *surf* model, in its initial iteration, is a model of the typical routines and displacements of those *9–5 commuters* on typical workdays. Having used all available evidence to estimate the activities of *commuters*, it becomes possible to create simulated estimates of the overall amounts of footfall that this group contributes to. There were, of course, clear discrepancies between the simulation of a single demographic group, and the real data that included a much broader range of individuals. Therefore, following discussions with local stakeholders another substantial group who are present in the case study area was added to the model; that of *retired people*. The inclusion of this new agent type substantially reduced the simulation error and paved the way to continuing this iterative processes, gradually increasing the number and diversity of groups who are modelled.

These results provide a potential avenue to better understand how urban spaces are being used in the absence of information about the behaviour of individuals in the study area. Although footfall data are available, these do not reveal information about the *individual people* who contribute to the aggregate footfall. Indeed, we would argue that it would be unethical to attempt to identify, and potentially begin to track, those individuals in the first place. In effect, therefore, the model provides a means to disaggregate the available footfall data by the demographics of the individuals who contribute to the aggregate counts. Agent-based modelling is a methodology that is ideally suited to this task as it offers a means of combining high resolution spatial data (in this case the census) with high resolution behavioural data (the time use survey). The model is used to marry these otherwise disparate datasets to create a more robust picture of daily urban dynamics.

There are, of course, some differences between the simulated and real data that remain. Fig. 7 illustrated these. The most notable difference is the reduction in footfall at approximately 15:00. This begs the question: who are the people who make up this extra footfall? This time corresponds closely with the time that children, both young – who will be collected by carers – and older – who are mostly unsupervised – will leave their schools. It is therefore extremely likely that this group (children and their carers) are the cause of this particular discrepancy. Interestingly, the inclusion of these groups in future iterations will not only require the addition of a new group (*schoolchildren*), but also the diversification of the existing groups as many *commuters* and *retired people* will be responsible for delivering children to and from school. There is evidence for this both in the time use survey and anecdotally following the discussions with stakeholders.

Regarding the location of the activities, it is also worth noting that some sensors, notably 1 and 14, suggest morning and afternoon peaks that are more indicative of *commuting* behaviour than others. This points to the possibility of identifying the most likely locations, as well as times, at which the different groups might be present. Fig. 8 focussed specifically on the locations of each of the sensors. This, again, has the potential to provide useful information about these non-commuting groups. If, for example, policy makers are concerned about the impacts of pollution on the elderly or young children, a method such as this could be used to provide evidence about the times and locations that the group will be most active. This information is otherwise extremely hard to gather using traditional sources such as surveys and censuses, and even more so using *big data* sources that are often biased towards certain age groups, potentially excluding the very young or very old. We see this as the main contribution of the paper; a means of simulating the daily spatio-temporal behaviour of different population groups, particularly for those whose activities are otherwise very difficult to interrogate.

### Caveats

There are a number of caveats that are important to note. Firstly, there are questions regarding the real-world footfall data that have yet to be resolved. For example, it is reasonable to assume that most smartphones will be counted by the sensors, so to estimate age or gender bias we can look for data on smartphone saturation within the population at large, at least if there is no specific bias in Wi-Fi usage between different groups. It is harder to estimate factors such as whether small numbers of people who, as an artefact of their activities, will trigger a sensor multiple times in an hour. Although there are some mechanisms that can be used to guard against this, e.g. by temporarily recording the unique identifier (MAC address) of the phone and ignoring it if it is counted more than once per hour, on modern smartphones the identifier changes regularly so this is not possible. Nevertheless, the footfall data are likely to represent a sufficiently accurate proxy for day time activity levels in the town. A few recent studies have shown that a bias in phone usage between demographic groups does not have a major effect on general travel patterns [38, 39].

Another potential problem is that although the footfall data are relatively recent, covering the period October 2015 to June 2017, the census data are from 2011. It is likely that some people will have moved or changed jobs in this time, but estimating the current commuting patterns in the area is beyond the scope of this work.

The intensity-based decision framework seems to work better when agents have at least one not-at-home regular activity with a fixed location that anchors their behaviour. If not, which is the case for retired people, too many agents do all their activities immediately in the morning. This does not reflect the patterns observed in the time use survey. The model seems to be more suited to dealing with commuters who have less flexibility and time (because they spend a large part of their day at work) to start shopping and doing leisure activities.

This model is somewhat unusual in comparison to other agent-based models in that it has not been empirically validated by comparing its outputs to data that it has not been calibrated on. Section 4 discussed this problem at length. Empirical (i.e. “fit to data” [34]) validation has not been undertaken for two reasons. Firstly, there are no data to validate the model against. The usual process of dividing up the real-world data into training and tests sets would not work here because the average daily weekday patterns would be identical in both the test and training sets (as evidenced by the extremely narrow confidence intervals produced in Figs. 6 and 8). Larger cities might have other *big data* sources that can be used to estimate the daytime population, such as public transport smart cards, but this is not the case in Otley. Secondly, empirical validation is of limited value here anyway. The aim of this paper is to explore whether the model might be able to provide new information about groups for whom activity data not currently exist, not (yet) to produce a robust empirical analysis. That said, attempts at face validation were made by presenting early results to the people who are the most familiar with the case study area. Although their observations are clearly anecdotal and the group itself was self-selected, their insights are still useful.

### Future work

Immediate future developments will improve the behaviour and diversity of the agents, the activity framework, and the routing algorithm. To begin with, the agents should be members of households. This is particularly important for modelling school children and their carers (this group was noted to be the most likely contributor to the difference between the model and real data). Data for households could be estimated from the census through spatial microsimulation [40, 41]. Doing activities with other household members would have an impact on the activity intensities of the agents. An extended model would also include a greater variety of leisure activities. The agents could also remember preferred locations for their flexible activities, which would then lead to a higher probability of that location hosting the activity in the future (i.e. the agents could build up preferences for certain locations). They could also learn to improve the logical order of their activities, so that their travel distance reduces. Furthermore, by accounting for different transportation modes, congestion could begin to emerge.

Although the model was calibrated, the process was performed manually by comparing the observed agent behaviour to the time use survey and footfall data and adjusting parameters accordingly (as discussed in Section 3.1). In the future, the calibration of the model parameters should be more automated. With the current mix of big data and traditional data, optimisation algorithms such as genetic algorithms, neural networks, or Bayesian approaches (e.g. [42]) could be considered. However, the most interesting approach to future calibration will include dynamic data assimilation techniques to make real-time data streams to forecast the ambient population [43] and update a running model accordingly.

## Conclusion

This paper has presented a somewhat unusual approach to agent-based modelling. The paper aims to produce new data about groups of people whose daily activities are absent in traditional data sets, but does this by first simulating a group for whom data are abundant, and then iteratively increasing the diversity of the simulated agent groups in order to reduce the difference between real and simulated data. As there are no empirical data with which the model can be validated, local stakeholders in the case study area were drawn on to interrogate the model outputs and compare them to their own experiences. Further model refinement and the inclusion of other agent types will shed more light on the spatio-temporal locations of different groups during the day. This could potentially be useful for policy makers who need to better understand the characteristics of the ambient population to reduce the impacts of problems such as air pollution or crime on particularly vulnerable groups.

## Electronic supplementary material

ESM 1 (DOCX 27 kb)

## Footnotes

1 The structure of links and nodes in a neural network, for example, is largely arbitrary and does not reveal any information about the structure of the system that it represents, regardless of how accurately it is able to make predictions.

2 The Otley Business Improvement District is an organisation that promotes the interest of local businesses and is therefore knowledgeable about patterns of use in the town centre and interested in the simulations of footfall.
