# Supplementary material for: Quantifying the ambient population using hourly population footfall data and an agent-based model of daily mobility
Source: Geoinformatica. 2019 Apr 27;23(2):201–20. doi: 10.1007/s10707-019-00346-1 (PMC7328437; doi:10.1007/s10707-019-00346-1)
Supplement: Supplementary file 1 — (DOCX 27 kb) [file 10707_2019_346_MOESM1_ESM.docx]

**Online Appendix. ODD+D**

**I Overview**

**I.i Purpose**

The purpose of the model is to get insight into the daily travel patterns of different demographic groups. The model has been developed for local policy makers in the first place, but can be useful for scientists in the fields of urban geography and transport planning. As a first test, we focus on the behaviour of two groups (commuters and retired people) on weekdays.

**I.ii Entities, state variables and scales**

Every demographic group is represented by a different agent class in the model. Currently, these are *commuters* and *retired* agents. The agents can perform different activities during the day. Each activity has an *intensity* – a need to do that activity – and the activity with the highest intensity at any given time is the one that drives an agent’s behaviour (although there is also a *minimal intensity decrease* for the present activity that prevents agents rapidly changing activities). The *total intensity* is made up of a *background intensity* and a *time intensity*, which differ by each activity. The background intensity increases gradually when the agent is not performing the activity, and decreases during the activity. Increments and decrements of the background intensity reflect the typical recurrence pattern and duration of activities. The time intensity reflects common times when agents do that activity, but includes personal preferences for every agent. *Fixed* activities have a fixed location and *flexible* activities have a variable location nearby the location of the previous activity. There are 7 activity types:

- being at home;
- working in an office (commuters only);
- shopping for food in supermarkets;
- leisure shopping (retired only);
- having lunch in a lunch place;
- going out in the evening (either for food or drinks); and
- doing sports.

The environment consists of buildings and roads, which are created using real GIS data representing the study area. Every building belongs to a type, and each type can be a suitable location for 1 or more activities. Agents travel along the roads from building to building in a continuous spatial environment, generated with GeoMASON classes. The model uses a time step of 5 minutes. The variables associated with the agents are given in Table A1, and the variables associated with the activities of an agent in Table A2.

**Table A1.** Variables associated with agents.

| **Variable** | **Value** | **Comment** |
| --- | --- | --- |
| location | state variable | Current location of an agent |
| moveRate | parameter value | Constant speed at which agents move. Can be different when travelling between home and work. |
| path | state variable | Route an agent will follow to location of next activity |
| cameras | state variable | List of footfall cameras agents pass by on their route (with time stamps) |
| activities | state variable | List of all activities for this agent class (see Table A2 for activity variables) |
| currentActivity | state variable | The current activity of the agent |
| rnd | random | Random variable that can shift the time intensity of activities to earlier or later hours, representing that some agents are more active during early or late hours. |
| rndLunchPref | random | Indicates if an agent likes to go for lunch in a cafe. |
| rndGoingOutPref | random | Indicates if an agent likes to go out for dinner or drinks. |
| rndSportPref | random | Indicates if an agent likes to do sports. |
| rndLunchShopPref | random | Indicates if a commuter agent prefers to do shopping for food during the lunch break. Preference for shopping after work is 1 – rndLunchShopPref. |
| rndOtherShopPref | random | Indicates if a retired agent likes to do leisure shopping. |
| home | random in home OA | Random building ID in the OA where the agent lives. |
| workPlace | random in work OA | Random building ID in the OA where the agent works (commuters only) |

**Table A2.** Variables associated with activities of an agent.

| **Variable** | **Value** | **Comment** |
| --- | --- | --- |
| activityType | state variable | Type of activity (e.g. *at home* or *working*). |
| intensity | state variable | Intensity to start this activity. Sum of backgroundIntensity and timeIntensity |
| backgroundIntensity | state variable | Part of intensity dealing with recurrence. |
| timeIntensity | state variable | Part of intensity dealing with the time of the day. |
| timeProfile | array of parameter values | Array of tuples with a time and a timeIntensity. Intermediate values get interpolated. Determines timeIntensity of the activity. Makes use of random preferences of agents for this activity, and for being active earlier or later, both as defined in Table A1. Is defined per activity type and agent class, but is agent specific due to the stochasticity included. |
| backgroundIncrease | parameter value | Determines how fast backgroundIntensity is increasing for an activity (not for the current activity). Different parameter values per activity type and agent class. |
| rndActivityIncrease | random | A random number in the range [0.8, 1.2], different for each agent, by which backgroundIncrease is multiplied |
| reduceActivityAmount | parameter value | Determines how fast backgroundIntensity is decreasing for the current activity. Different parameter values per activity type and agent class. |
| minimumIntensityDecrease | parameter value | Determines how long it takes to finish an activity. The intensity has to decrease at least by this value before another activity can start. Different values for each activity type. |
| highestActivityThreshold | Constant  = 0.75 | After satisfaction of the currentActivity, a new activity will start if the intensity for that activity is higher than this threshold (or anyway if the intensity of the currentActivity drops below zero). |

**I.iii Process overview and scheduling**

Initialisation is discussed in section III.ii. The following outlines how the agents update their activity during every time step:

- The background intensity of all activities is increased, except for the current activity.
- Check the status of the current activity. There are 3 options:
  - 1. Initialisation. In the first time step after the agent has decided to start a new activity, the location of that activity will be determined. The location of activities with a non-fixed (*flexible*) location (all except *being at home* and *working*) is chosen randomly from all possible nearby locations. This is done within a search radius which is initialised in proportion to the size of the study area. For the Otley area, the initial value is 215m^[[1]](#footnote-1)^. If no suitable location can be found for the activity, the radius is iteratively doubled until one or more buildings are available. If more than one building is found, the location for the activity is randomly assigned to one of the buildings. Next, the shortest path towards the chosen building is determined using the A* shortest path algorithm (as implemented in GeoMASON) and then stored. Finally, the agent starts travelling.
  - 2. Travelling. Move along the stored path with a constant speed.
  - 3. Perform activity. If the destination has been reached in an earlier time step, then the background intensity for this activity will decrease.
- If the background intensity of the current activity has decreased more than the minimumIntensityDecrease for that activity, then:
  - Rank all other activities by their intensity, but only include those with timeIntensity > 0.
  - Keep on doing the current activity IF the intensity of none of the others is above the highestActivityThreshold AND the intensity of the current activity is still higher than 0.
  - ELSE, change the current activity to the one with the highest rank.

**II Design concepts**

**II.i Theoretical and empirical background**

The model concepts are loosely based on frameworks to model the decisions of individual agents, like the PECS model [Schmidt, 2000; Urban, 2000]. More information on the general concepts can be found in the Introduction section of the paper. There is bounded rationality and no foresight in the model. Agents are only aware of the study area and do not make any plans, other than those that are required to satisfy their immediate goals (i.e. plan a route to a destination). The behaviour of the agents should empirically reproduce the observations present in the 2014-15 UK Time Use Survey.

**II.ii Individual decision-making**

Agents decide themselves which activities they do based on the intensities for those activities. There is not an explicit objective or goal to be reached. Exogenous state variables, social norms and cultural values do not influence the decision-making process. Spatial and temporal aspects are important. Activities with a *flexible* location are more likely to happen nearby the location of the previous activity. The time of the day has a strong effect on the intensities of the activities. Uncertainty is included since the model is run many times and stochasticity will change the results slightly every time.

**II.iii Learning**

Learning is not yet included in the model. The agents do not change their behaviour based on their experiences (e.g. optimise the order of their activities in order to have shorter total travel times per day). They also do not have preferences for specific shops, restaurants, or sports locations although this will be improved in future work.

**II.iv Individual sensing**

Individuals sense the need to start specific activities, depending on how much time they have already spent on those activities in the recent past. They know the locations of their fixed activities and can find a place to do their flexible activities nearby their present location. Mechanisms to gather information are not explicitly modelled.

**II.v Individual prediction**

The future condition of the activities of agents largely depends on the past condition of which activities they have been recently doing, and on the random initialisation of their preferences.

**II.vi Interaction**

There are no interactions between the agents. They are not aware of each other and do not compete for resources. Buildings and road sections can contain an infinite number of agents if necessary. A future version of the model should include interactions between the agents (e.g. they could belong to a household) and have realistic traffic and congestion that determines the speed to reach locations.

**II.vii Collectives**

There are no collectives or aggregations of agents.

**II.viii Heterogeneity**

All agents have their own preferences to do specific activities as well as their own preferences to start the day earlier of later, which is all reflected in their personal time profile for each activity. Background intensities also increase in a different way for each agent, leading to a personal recurrence pattern.

**II.ix Stochasticity**

Stochasticity is important to determine the intensity values (both time intensity and background intensity) of the different agents as described in the previous section. Buildings for *fixed* activities are randomly chosen within an Output Area, and buildings for *flexible* activities are randomly chosen within all nearby buildings of the right category for that activity. An overview of all random values can be found in Tables A1 (for agent properties) and A2 (for activity properties).

**II.x Observation**

Tables with information on the timing of all activities are stored for a random sample of agents, and are used for calibration. The frequency of the number of agents of each class doing a specific activity can be extracted from these tables to validate the agent behaviour. Furthermore, a table with the total number of agents walking past footfall cameras per hour is stored, and can be used as a validation of the results in both space and time.

**III Details**

**III.i Implementation details**

The model has been implemented in Java and Scala, and makes use of agent classes and GIS functions of the Java-based (Geo)MASON platform. The code can be accessed on <https://github.com/nickmalleson/surf>.

**III.ii Initialisation**

During initialisation, agents are created for each agent class. The number of agents at home and at work in each Output Area is based on the 2011 Census data. All commuters who are commuting inside the study area are represented as individual agents, as are all retired people. The model starts at midnight with all agents being at home. The background intensity for *being at home* is assigned a random value in the interval [0.5, 1] for commuters and [0.6, 1] for retired agents (determined after calibration). Background intensities for all other activities start with a value of 0. The time intensities of all activities are those for midnight for that agent (including stochasticity to make every agent’s personal time profile).

Building locations were obtained from Ordnance Survey Mastermap Topography Layer data and building function data (different types of shops, food and drink places, and sport venues) come from OpenStreetMap, after which both are pre-processed and combined in ArcGIS and R. Buildings that do not have a specific function are split up into small and large buildings. *Being home* and *working* are the only activities with a fixed location that never changes for the agents. All agents are assigned home locations randomly in small buildings from within their home output areas (OAs), and commuter agents are assigned work locations in the same way from within their work OA. The actual home and work locations in reality will be similar as OAs are typically very small (covering on average 0.3 km^2^ in the study area).

**III.iii Input data**

See above in section III.ii.

**III.iv Submodels**

There are no submodels.

**References**

Schmidt B (2000) The Modelling of Human Behaviour. SCS Publications, Erlangen, Germany

Urban C (2000) PECS: A reference model for the simulation of multi-agent systems. In: Suleiman R, Troitzsch KG, Gilbert N (eds) Tools and Techniques for Social Science Simulation, Physica-Verlag, Heidelberg, pp 83–114

1. When the simulation starts, a search radius of 215m is calculated by the model. This balances the efficiency of a using a small search radius that does not return too many objects with the inefficiency of having to increase the radius and begin the search again if no objects are found initially. [↑](#footnote-ref-1)
